# Multisensory integration involved in the body perception of community-dwelling older adults

**DOI:** 10.1038/s41598-021-81121-x

**Published:** 2021-01-15

**Authors:** M. Hide, Y. Ito, N. Kuroda, M. Kanda, W. Teramoto

**Affiliations:** 1 Department of Psychology, Graduate School of Social and Cultural Sciences, Kumamoto University, 2-40-1 Kurokami, Kumamoto, 860-8555 Japan; 2 Department of Psychology, Faculty of Letters, Kumamoto University, 2-40-1 Kurokami, Kumamoto, 860-8555 Japan; 3 Department of Psychology, Graduate School of Humanities and Social Sciences, Kumamoto University, 2-40-1 Kurokami, Kumamoto, 860-8555 Japan

**Keywords:** Human behaviour, Cognitive neuroscience, Perception

## Abstract

This study investigates how the multisensory integration in body perception changes with increasing age, and whether it is associated with older adults’ risk of falling. For this, the rubber hand illusion (RHI) and rubber foot illusion (RFI) were used. Twenty-eight community-dwelling older adults and 25 university students were recruited. They viewed a rubber hand or foot that was stimulated in synchrony or asynchrony with their own hidden hand or foot. The illusion was assessed by using a questionnaire, and measuring the proprioceptive drift and latency. The Timed Up and Go Test was used to classify the older adults into lower and higher fall-risk groups. No difference was observed in the RHI between the younger and older adults. However, several differences were observed in the RFI. Specifically, the older adults with a lower fall-risk hardly experienced the illusion, whereas those with a higher fall-risk experienced it with a shorter latency and no weaker than the younger adults. These results suggest that in older adults, the mechanism of multisensory integration for constructing body perception can change depending on the stimulated body parts, and that the risk of falling is associated with multisensory integration.

## Introduction

Body perception is established through the integration of information from different sensory modalities, especially the visual, somatosensory, and proprioceptive systems. The rubber hand illusion (RHI) is one of the most commonly used experimental paradigms for investigating this process. In a typical RHI experiment, a rubber hand is placed on a table in front of a participant next to their real hand, which is placed out of view behind a screen. After a few minutes of spatiotemporally synchronous stimulation of both the rubber and real hands with a paintbrush, during which the participant observes the rubber hand, the participant feels as if the rubber hand is part of their own body. When this occurs, they often perceive the position of the hidden real hand to be closer to the rubber hand than its actual physical position^1^, and can experience a lower skin temperature in the real hand^2^. In contrast, asynchronous stimulation reduces the RHI, which indicates that the temporal congruency between the visual and somatosensory inputs is key to an individual constructing body ownership and localizing their own body parts^1^. Some studies have reported that the RHI can also be induced without tactile stimulation, which suggests that visual information has a stronger influence on the construction of the representation of the body^3^.

Recent evidence has suggested that the multisensory integration process is not constant across a person's lifespan and can change with increasing age. Studies have generally reported that older adults exhibit enhanced multisensory integration (see^4^ for a review). Several different hypotheses have been put forward to explain this, such as general cognitive slowing, inverse effectiveness, increased temporal window of integration, and deficits in attentional control^4^. However, regarding body ownership and localization of the hand, most studies have shown that there are no or few age-related differences. For example, Campos et al.^5^ compared the RHI between older adults who were 65 years old or over (mean age: 73 years) and younger adults (mean age: 24 years). The authors reported no effect of age on any measures, including proprioceptive drift, skin temperature (a physiological measure), or subjective ratings of body ownership. No or weak differences between older and younger adult groups have been found in several other studies^6–8^; however, in the studies by Kállai et al.^9^ and Graham et al.^10^, in which adults below 60 years old had participated, a decrease in subjective rating of body ownership^9,10^ and an increase in proprioceptive drift^10^ with increasing age has been reported. Thus, the multisensory processing involved in body perception seems to be relatively stable even in late adulthood. This differs from other types of perceptual processes that are established using multisensory information, such as speeded target detection, localization, and temporal discrimination^4^.

The first aim of the present study was to further investigate body perception in older adults by focusing on two new aspects: the generalizability of the illusion to another body part (the foot) and the association of the illusion with the risk of falling. Studies have shown that illusions similar to the RHI can extend to other parts of the body^11,12^, including the face^13^, tongue^14^, and head^15^. More recent studies have shown that this can also extend to the lower leg or foot^16,17^. Flögel et al.^17^ compared the RHI and rubber foot illusion (RFI) and reported that comparable illusions could be induced, irrespective of the body part. These studies suggest that the multisensory integration of the visual, somatosensory, and proprioceptive information that evokes the RHI is not limited to the generation of the representation of the hand, and that it can also generalize to the representation of other body parts. Nevertheless, the principle of multisensory integration holds that the brain integrates information from several sensory modalities based on the relative reliability (or precision) of each input^18,19^. As we age, almost all sensory signals are reduced compared with those of younger adults. Proprioceptive information from the lower limb may deteriorate much more compared to that from other body parts as well as information from other sensory modalities, because studies have shown that there is a decrease in gait function with increasing age^20^. Therefore, the present study investigated whether there are differences in the RHI and RFI between younger and older adults.

The second aim was to investigate the association between the illusions and an individual’s risk of falling. Recent studies have shown that age-related changes in the multisensory integration process are related to this risk. In the audio-visual domain, Setti et al.^21^ reported that the double flash illusion occurred with a wider range of time differences between the auditory and visual stimuli for older adults with a history of falls than for those without one; this effect was reduced after a balance training intervention for fall-prone older adults^22^. The alteration of the multisensory processing associated with falls or the risk of falling is also reported in the visuo-somatosensory domain. Mahoney et al.^23^ showed that older adults who exhibited multisensory enhancement had a less stable posture than those who did not exhibit it (however, see^24^: the authors showed the opposite results). Teramoto et al.^25^ reported that the range of the visual enhancement of tactile detection expanded sagittally for older adults with decreased gait and balance functions. These studies suggest that, compared to healthy older adults, older adults with a higher fall risk might exhibit altered multisensory integration in the creation of their body representations.

## Methods

### Participants

Twenty-eight community-dwelling older adults (mean age: 78.79 ± 6.29 years, minimum = 65, maximum = 88; two men) were recruited via a local club for the aged and the personal connections of supporters. All participants scored more than 24 on the Mini-Mental State Examination (MMSE)^26,27^, indicating that they had no cognitive impairment. They did not have any type of dementia, depression, stroke, parkinsonism, or tactile impairments, and were not currently receiving treatment with neuroleptics. They performed the tasks while subjectively having the clearest view of both the rubber hand and foot: The eyes of 11 participants were corrected by convex or bifocal glasses; those of the others were not. Additionally, six participants reported defects in vision in either eye (e.g., macular degeneration, cataract, or glaucoma). The visual acuity for each participant when using both eyes is shown in Table 2. A group of younger participants was also recruited, which included 25 undergraduate and graduate students (mean age: 22.44 ± 1.83 years, minimum = 21, maximum = 28; 12 men) who had normal or corrected-to-normal visual acuity. This study was conducted according to the principles of the Declaration of Helsinki and was approved by the Ethics Committee of the Graduate School of Social and Cultural Sciences, Kumamoto University. The participants provided written informed consent to participate in this study and to publish their accompanying images in an online open-access publication before the commencement of the experimental sessions.

### Assessment of the cognitive, sensory, and sensory-motor functions of the younger and older adults

The Trail Making Tests A and B (TMT-A and TMT-B) were used to assess the participants’ executive functions, including attentional control and task switching^28–31^. The Timed Up and Go (TUG) test^32^ was used to assess the participants’ sensorimotor functions related to gait (i.e., the fall risk). This test is commonly employed as a clinical tool to identify older adults who are at risk of falling^32^. During the TUG test, the participants were asked to stand up from a standard chair with a seat height of approximately 40 cm, walk as quickly as possible to a marker placed at 3 m, turn around the marker, walk back to the chair, and sit down. The time was measured with a stopwatch from the point of standing up to being re-seated. We also assessed the older participants’ handgrip strength, which is reported to be related to lower-limb strength^33,34^. Each participant performed this test twice with their right and left hands. The average of the stronger scores for each hand was used as the representative value.

The amount of daily physical activity is also related to the change in the sensorimotor ability of older adults^35^. Therefore, we asked the older participants to wear a pedometer for three consecutive weeks, and the average number of steps per day was used to assess daily physical activity. All participants’ visual acuity, which is equivalent to the reciprocal of the minimum resolvable visual angle, was assessed at viewing distances of 5 m and 0.4 m with both eyes open using Landolt C charts. The tactile sensitivity of all digits of the right hand and foot was measured using the Von Frey Touch Test (Semmes–Weinstein monofilaments; North Coast Medical Inc., Morgan Hill, CA, USA). This test measures the minimum force required for participants to feel touch on their skin.

### Experimental setups of the RHI and RFI

**Figure 1:**
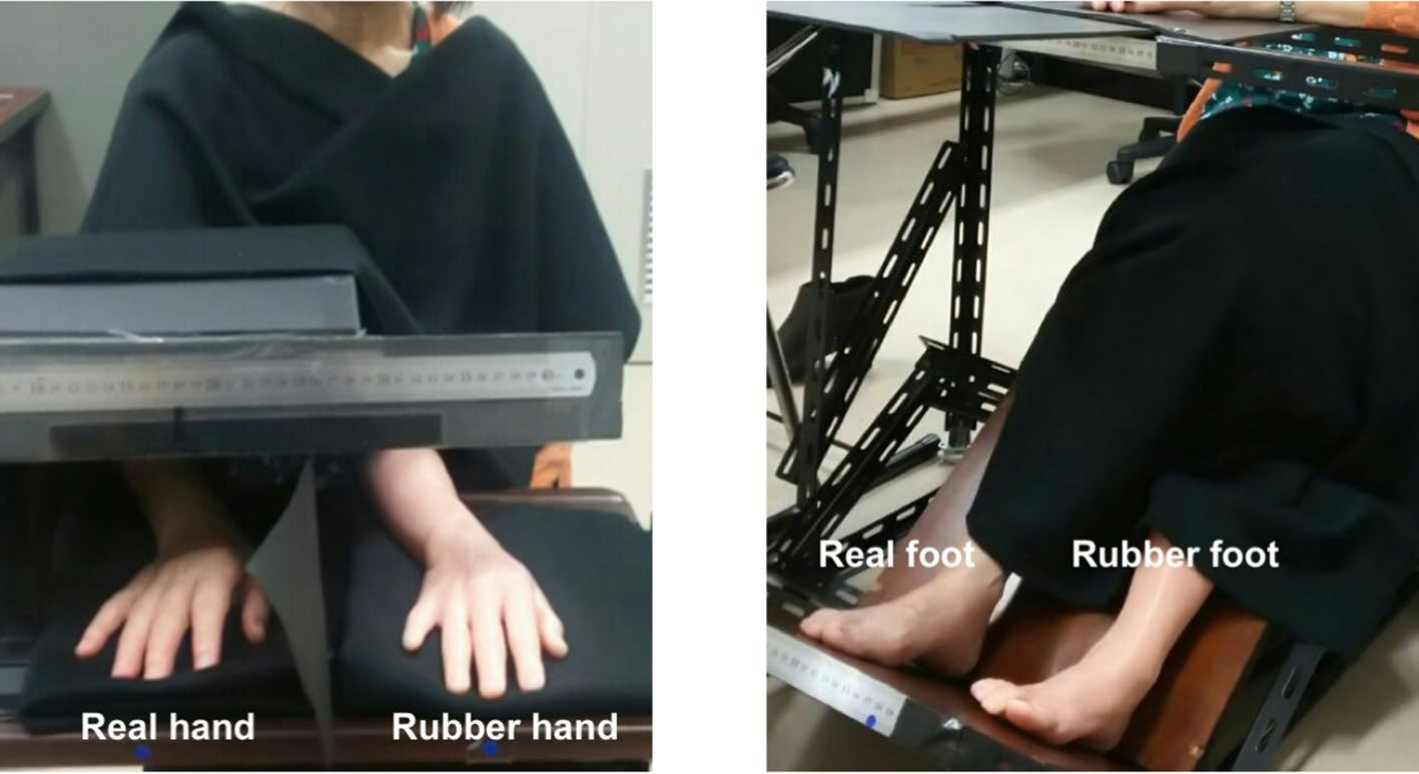
Experimental setups of the rubber hand illusion (left) and the rubber foot illusion (right), respectively. The participant’s right hand and foot was placed 20 cm to the right of the artificial counterpart on the equipment. A ruler was attached to the front of the equipment to measure the perceived position of the participant’s real digit.

The participant was seated on a chair in front of a table. During the RHI induction phase, their right hand was placed on the table and was occluded by a black-colored box (Fig. 1, left panel). A custom-made silicon right hand was placed 20 cm to the left of the participant's right hand. The participant’s upper arm and shoulder and the lower part of the silicon arm were covered by black fabric to give the participant the impression that the rubber hand was their real hand.

For the RFI induction phase, the participant’s right foot was placed on a custom-made footrest (Fig. 1, right panel). A custom-made silicon right foot was placed 20 cm to the left of the participant's right foot. The participant’s legs and the leg part of the silicon model were occluded by black fabric to give the impression that the rubber foot was their real foot.

### Procedure

Four conditions were formed based on the combination of two stroking styles (synchronous [SYNC] and asynchronous [ASYNC]) and the two body parts (hand and foot). The tactile stimulation was performed manually. In the SYNC condition, identical digits of the rubber and real body parts were synchronously stroked three times in sequence from the first to the fifth digit at approximately 1 Hz for 3 min. The stroke was delivered between the first and second joints of the digit in the distal direction. In the ASYNC condition, the stroke was alternated between the real and rubber body parts. After three sets of alternate strokes between the identical digits of the real and rubber body parts, the next pair of digits were stimulated in the same way. The stroke started from the first digit and sequentially moved to the fifth digit. This sequence continued for 3 min.

In the RHI condition, the participants placed their right hand inside the occluding box and were asked to keep looking at the rubber hand. In the RFI condition, the participants removed their shoes and socks in order to receive the brushstrokes on their skin and they placed their foot in a specific position. In all conditions, the felt position of the middle digit of the participant's right body part was measured first (see the Measures subsection for more detail). During the induction phase, the participants were instructed to keep looking at the rubber body part without moving their real one and to say “yes” when they felt as if the rubber hand was a part of their own body. Immediately after the stimulation, the post-location judgment of their real body part was obtained and, finally, the participants were asked to complete the questionnaire in a light and relaxing atmosphere. The order of the RHI and RFI was counterbalanced; that of the SYNC and ASYNC was also counterbalanced to reduce any order effect.

### Measures

#### Latency

Latency was measured from the point at which the participants started to receive tactile stimulation to when they experienced the RHI or RFI. The percentage occurrence of the illusions was defined as the percentage of participants who reported the RHI or RFI within 3 min of the induction phase. These measures were performed to investigate whether and when the participants subjectively experienced the illusions. The latency data were obtained only from those who experienced the illusion within 3 min.

#### Questionnaire

A questionnaire developed by Longo et al.^36^ was used to assess how the rubber body part was integrated into the participants’ body representations. For the present study, the items were translated from English into Japanese. The questionnaire comprises nine items divided into three categories: ownership (Q1–4), which is the feeling of ownership of the rubber hand or foot; localization (Q5–7), which is related to the feeling of where their own hand or foot is and where they experience touch; and agency (Q8–9), which is related to the feeling of being able to move the rubber hand or foot and control it. The participants were required to respond on a Likert scale ranging from 1 (strongly disagree) to 7 (strongly agree). The questionnaire for the RHI is shown in Table 1. For the RFI, the word “hand” in the RHI questionnaire was replaced with the word “foot”.

**Table 1:** Questionnaire items for the rubber hand illusion experiment.

Q1. It seems as if I saw my own hand rather than the rubber hand
Q2. The rubber hand began to resemble my own (real) hand
Q3. I felt as if the rubber hand belonged to me
Q4. I felt as if the rubber hand were my hand
Q5. It felt as if my real hand was drifting toward the rubber hand
Q6. It appeared as if the rubber hand was drifting toward my real hand
Q7. It seemed as if I was feeling the paintbrush touch the location where I saw the rubber hand touched
Q8. It seemed like I could have moved the rubber hand if I had wanted to
Q9. It seemed like I was in control of the rubber hand

*Note* In the questionnaire for the rubber foot illusion, the word “hand” was replaced with the word “foot”.

#### Proprioceptive drift

The proprioceptive drift was used to quantify the reconstructed body representations. It was defined as the distance of the felt position of the middle digit of the hidden right hand (foot) between the pre- and post-RHI (RFI) induction phase. The origin of the measurement was the position of the real hand. Positive values indicated a drift toward the rubber body part from the real one, while negative values indicated a drift away from the rubber body part. Before and after the induction phase, the room light was turned off and the experimenter moved a light-emitting diode (LED) device from the far right toward the midline. It was moved along the box edge in the RHI experiment and along the footrest edge in the RFI experiment. The participants were asked to say “stop” when they felt that the LED device was over their middle digit. The LED device was located approximately 30 cm above the stimulated hand and 65 cm above the stimulated foot. The initial position of the LED device was randomly shifted trial by trial to avoid possible learning effects. Additionally, to investigate the variability in the participants’ proprioceptive sense, the absolute difference in the perceived positions of the middle digit between the SYNC and ASYNC conditions before the illusion induction was calculated.

### Statistical data analysis

Statistical analyses were conducted using IBM SPSS for Mac, version 25 (IBM Corp., Armonk, NY, USA). For the questionnaire data, a single principal component analysis (PCA) was performed that included all the data, irrespective of the age group and stroking condition. The Kaiser–Meyer–Olkin measure of sampling adequacy was 0.931 and the Bartlett’s test of sphericity was significant (χ^2^(36) = 2866.34, *p* < 0.001), which indicated the validity of the PCA for the current data. Only one component was extracted, which accounted for 83.49% of the observed variance. Thus, the average score of all nine items was calculated as “subjective rating” and analyzed. The Shapiro–Wilk tests revealed that all the data, except for latency, were not normally distributed. Therefore, these data were analyzed using nonparametric methods. Regarding latency, only the data of participants who experienced the illusions in the SYNC condition were analyzed, as most participants did not report experiencing the illusions in the ASYNC condition.

The first stage of the analysis focused on the differences between the younger and older adults. Demographic information, including the variability in proprioceptive sense, was analyzed using the Mann–Whitney *U* tests. The group differences were analyzed using the chi-square tests for the percentage occurrence of illusions, and the Mann–Whitney *U* tests for latency, subjective rating of illusion, and proprioceptive drift measures. Additionally, the effect of stroking (SYNC vs. ASYNC) within each group was analyzed for the percentage occurrence and subjective rating of illusion, and proprioceptive drift measures using the same tests.

In the second stage of the analysis, the older adults were classified into two groups based on their TUG scores to investigate the relationship between body representations and fall risk. After the TUG scores were listed in ascending order, the top ten older adult participants (who had shorter TUG times) were assigned to a low fall-risk group (LOW_fall-risk_) and the bottom ten older adults (who had longer TUG times) were assigned to a high fall-risk group (HIGH_fall-risk_). The demographic data were compared between these two older adult groups using Mann–Whitney *U* tests. Using the data of the younger adult group as baseline, the differences between the groups were analyzed using the Fisher’s exact tests for percentage occurrence of illusions, and the Kruskal–Wallis tests for latency, subjective rating, and proprioceptive drift. For multiple comparison between the groups, the stepwise-stepdown procedure^37^ was used. The effect of stroking within each group was analyzed similar to that in the first stage of the analysis.

## Results

### Comparison between the older and younger adults

Table 2 shows the demographic information and results of the cognitive and perceptual tests of the older and younger participants. The older adults exhibited significantly larger TUG, TMT-A, and TMT-B scores; and lower visual acuity and sensitivity to tactile stimuli, compared to the younger adults (|*Z*|> 3.348, *p* < 0.001, *r* > 0.460). For variability in the proprioceptive sense, no significant difference was observed between the age groups for the hand (older adults: *|M|*= 2.21 cm, *SD* = 1.68 cm; younger adults: *|M|*= 1.57 cm, *SD* = 1.40 cm; *Z* = − 1.525, *p* = 0.127, *r* = 0.210), or for the foot (older adults: *|M|*= 2.21 cm, *SD* = 1.68 cm; younger adults: *|M|*= 3.12 cm, *SD* = 2.60 cm; *Z* = 0.579, *p* = 0.562, *r* = 0.080).

**Table 2:** Demographic information, the cognitive and perceptual abilities of the younger and older adults, and the results of Mann–Whitney *U* tests.

Age group	Young (*N* = 25)		Old (*N* = 28)		*Z* value	*P* value	Effect size (*r*)
Age	22.44 (1.83)		78.79 (6.29)		− 6.271	< .001	.861
Visual acuity (5 m)	1.05 (0.26)		0.78 (0.23)		3.348	.001	.460
Visual acuity (0.4 m)	1.38 (0.15)		0.52 (0.24)		6.060	< .001	.832
Tactile sensitivity (Hand)	32.99 (17.46)		82.61 (73.24)		− 3.335	< .001	.458
Tactile sensitivity (Foot)	56.86 (79.01)		431.17 (675.99)		− 4.144	< .001	.569
MMSE	–		28.07 (1.82)				
TUG	5.74 (0.83)		7.35 (1.65)		− 4.028	< .001	.553
Physical activity (# of steps)	–		4757 (2614)				
Grip force (kg)	–		21.94 (4.87)				
TMT-A	45.95 (10.37)		77.71 (29.12)		− 5.132	< .001	.705
TMT-B	56.01 (12.93)		128.14 (49.31)		− 5.890	< .001	.809

*Note* Visual acuity is the reciprocal of the minimum resolvable visual angle as measured using Landolt C charts. Tactile sensitivity is the minimum force (mg) with which participants can detect touch, which was measured using Von Frey monofilaments. The values in parentheses are standard deviations. The three rows on the right are the results of Mann–Whitney *U* tests to investigate the difference between the two age groups.

*TUG* timed up and go test, *MMSE* mini-mental state examination, *TMT-A* trail making test A, *TMT-B* trial making test B.

Significantly more participants experienced the RHI and RFI in the SYNC condition (old_hand: 60.7%; young_hand: 56.0%; old_foot: 64.3%; young_foot: 64.0%) than in the AYSNC condition (old_hand: 32.1%; young_hand: 16.0%; old_foot: 32.1%; young_foot: 24.0%), irrespective of the age group (old_hand: χ^2^(1) = 4.595, *p* = 0.032; old_foot: χ^2^(1) = 5.793, *p* = 0.016; young_hand: χ^2^(1) = 8.681, *p* = 0.003; young_foot: χ^2^(1) = 8.117, *p* = 0.004). Thus, the typical pattern regarding the effect of stroking was observed in our experimental setups, irrespective of body parts and age groups. There was no statistical difference in these values between the age groups (hand_SYNC: χ^2^(1) = 0.121, *p* = 0.728; foot_SYNC: χ^2^(1) = 0.001, *p* = 0.983; hand_ASYNC: χ^2^(1) = 1.860, *p* = 0.173; foot_ASYNC: χ^2^(1) = 0.432, *p* = 0.511).

**Figure 2:**
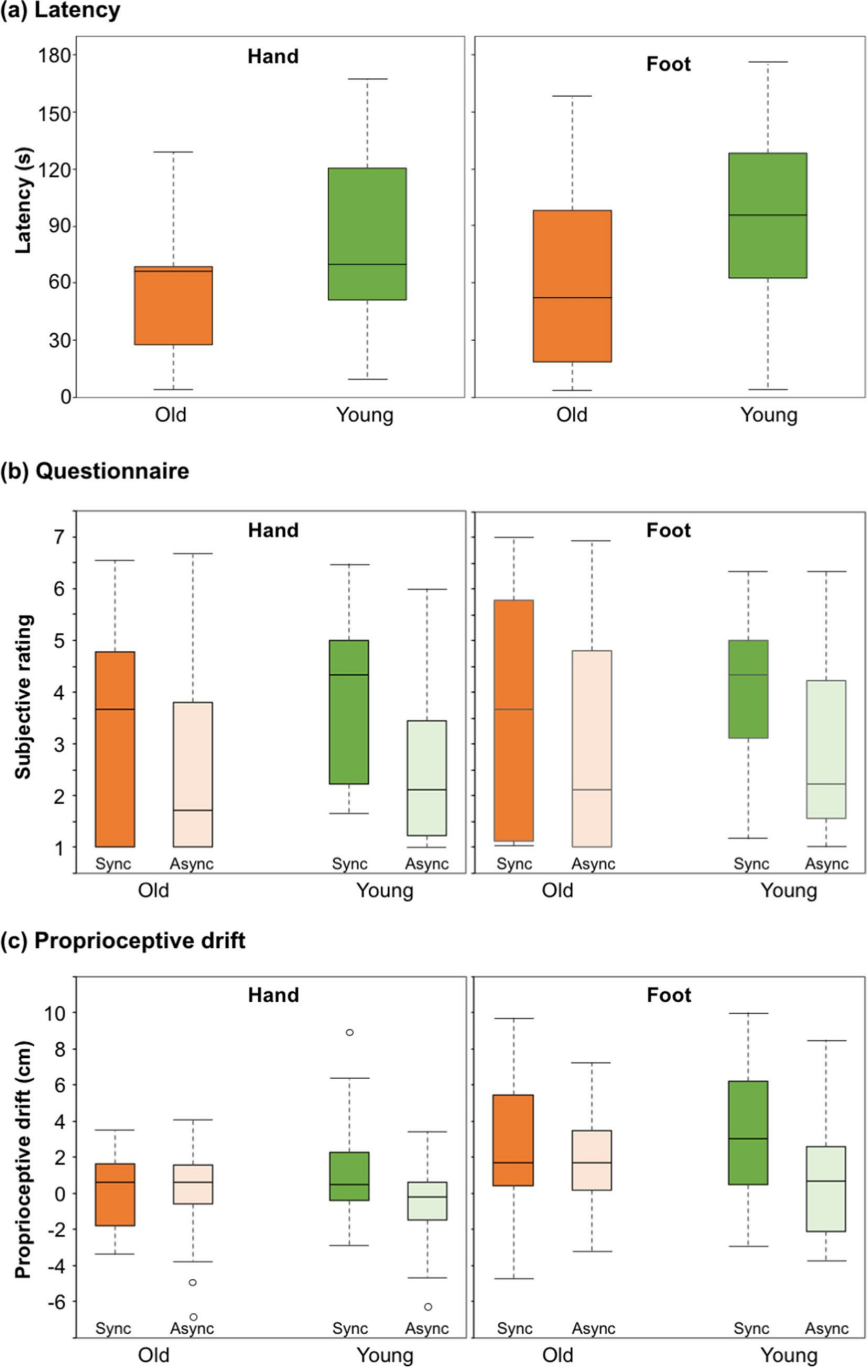
Boxplots of each measure in the younger and older adults. (**a**) Latency in the synchronous condition. (**b)** Subjective rating of illusion in the synchronous and asynchronous conditions. The subjective rating is the average of all nine questionnaire items. (**c**) Proprioceptive drift. The origin of the proprioceptive drift measurement was the position of the real hand. Positive and negative values indicate a drift toward and away from the rubber body part, respectively. The middle line, and upper and lower limits of the boxplot indicate the median, 75th, and 25th percentile. The error bars represent the maximum or minimum values where all data in the given condition fall between 1.5 times above and below the interquartile range. However, the bars represent 1.5 times above and below the interquartile range where there are outliers (blank circle) that fall beyond 1.5 times above and below the interquartile range.

As for latency (SYNC condition only; Fig. 2a), the younger adults took longer (RHI: 85.64 s ± 53.34 s [standard deviation]; RFI: 92.94 s ± 47.21 s) to perceive the illusions than older adults (RHI: 56.29 s ± 33.90 s; RFI: 64.00 s ± 52.80 s), but the differences were not significant in either the RHI (*Z* = 1.577, *p* = 0.116, *r* = 0.280) or RFI (*Z* = 1.822, *p* = 0.071, *r* = 0.312). Figure 2b,c show the results of the younger and older adults’ subjective rating and proprioceptive drift in the RHI and RFI. When directly comparing the younger and older adults, no significant difference was observed for either measure in the hand SYNC condition (subjective rating: *Z* = 1.224, *p* = 0.221, *r* = 0.168; proprioceptive drift: *Z* = 0.811, *p* = 0.417, *r* = 0.111), foot SYNC condition (subjective rating: *Z* = 0.856, *p* = 0.392, *r* = 0.118; proprioceptive drift: *Z* = 0.856, *p* = 0.392, *r* = 0.118), hand ASYNC condition (subjective rating: *Z* = 0.712, *p* = 0.447, *r* = 0.098; proprioceptive drift: *Z* = − 1.810, *p* = 0.070, *r* = 0.249), or foot ASYNC condition (subjective rating: *Z* = 0.261, *p* = 0.794, *r* = 0.036; proprioceptive drift: *Z* = − 1.470, *p* = 0.141, *r* = 0.202). In the younger adults, the effect of stroking was observed in both measures; i.e., a higher subjective rating and larger proprioceptive drift were observed in the SYNC condition compared to the ASYNC condition in both the RHI (subjective rating: *Z* = − 3.501, *p* < 0.001, *r* = 0.700; proprioceptive drift: *Z* = − 2.715, *p* = 0.007, *r* = 0.543) and RFI (subjective rating: *Z* = − 3.135, *p* = 0.002, *r* = 0.627; proprioceptive drift: *Z* = − 3.086, *p* = 0.002, *r* = 0.617). In the older adults, a significant effect of stroking was observed in the RHI for the subjective rating of illusions (*Z* = − 2.510, *p* = 0.012, *r* = 0.474), but not the proprioceptive drift (*Z* = 0.205, *p* = 0.838, *r* = 0.039). However, no significant effect of stroking was observed in the RFI for either the subjective rating of illusion (*Z* = − 1.611, *p* = 0.107, *r* = 0.304) or proprioceptive drift (*Z* = − 1.150, *p* = 0.250, *r* = 0.217). These results suggest that the younger and older adults experienced a comparable magnitude of illusion in the RHI; however, there were some differences in the RFI, as shown in the effect of stroking.

### Comparison between older adults with high risk and low risk of falling

Table 3 shows the demographic information and results of the cognitive and perceptual tests for the LOW_fall-risk_ and HIGH_fall-risk_ groups. Older adults with a higher risk of falling exhibited significantly slower TUG times, a lower amount of physical activity, and a smaller grip force than those with a lower risk of falling (|*Z*|> 2.343, *p* < 0.019, *r* > 0.524). The TMT-A score was also marginally significant: the HIGH_fall-risk_ group took longer to complete the task than the LOW_fall-risk_ (*Z* = 1.817, *p* = 0.075, *r* = 0.406). No significant differences were observed between the two groups for other demographic information or in the results of the cognitive and perceptual tests (|*Z*|< 1.512, *p* > 0.143, *r* < 0.338). No significant difference was observed in the variability in the proprioceptive sense for the hand (HIGH_fall-risk_: |*M*|= 2.49 cm, *SD* = 1.47 cm; LOW_fall-risk_: |*M*|= 2.58 cm, *SD* = 2.10 cm; *Z* = − 0.189, *p* = 0.853, *r* = 0.042) or the foot (LOW_fall-risk_: |*M*|= 3.09 cm, *SD* = 2.38 cm; HIGH_fall-risk_: |*M*|= 2.59 cm, *SD* = 1.43 cm; *Z* = − 0.644, *p* = 0.529, *r* = 0.144) either.

**Table 3:** Demographic information, the cognitive and perceptual abilities of LOW_fall-risk_ and HIGH_fall-risk_ groups of older adults, and the results of Mann–Whitney *U* tests.

TUG based grouping	Low fall risk (*N* = 10)		High fall risk (*N* = 10)		*Z* value	*P* value	Effect size (*r*)
Age	78.10 (7.08)		81.80 (3.79)		1.067	.315	.239
Visual acuity (5 m)	0.77 (0.30)		0.81 (0.20)		0.420	.684	.094
Visual acuity (0.4 m)	0.54 (0.36)		0.52 (0.14)		0.668	.529	.149
Tactile sensitivity (Hand)	69.96 (47.82)		101.68 (99.51)		0.416	.684	.093
Tactile sensitivity (Foot)	442.96 (654.25)		623.92 (902.40)		0.681	.529	.152
MMSE	28.50 (2.17)		27.90 (1.52)		− 1.205	.247	.269
TUG	5.87 (0.38)		9.14 (1.29)		3.781	< .001	.854
Physical activity (# of steps)	4812 (937)		4454 (4188)		− 2.343	.019	.524
Grip force (kg)	25.40 (3.53)		18.34 (5.07)		− 2.913	.002	.651
TMT-A	65.30 (21.51)		91.30 (36.52)		1.817	.075	.406
TMT-B	124.50 (71.48)		141.90 (29.70)		1.512	.143	.338

*Note* Visual acuity is the reciprocal of the minimum resolvable visual angle as measured using Landolt C charts. Tactile sensitivity is the minimum force (mg) with which participants can detect touch, which was measured using Von Frey monofilaments. The values in parentheses are standard deviations. The three rows on the right are the results of Mann–Whitney *U* tests to investigate the difference between the low fall-risk group (LOWfall-risk) and the high fall-risk group (HIGHfall-risk).

*TUG* timed up and go test, *MMSE* mini-mental state examination, *TMT-A* trail making test A, *TMT-B* trial making test B.

The percentage occurrence of illusions for the SYNC condition in the LOW_fall-risk_ and HIGH_fall-risk_ groups were 70.0% and 60.0% in the RHI, respectively, and 50.0% and 70.0% in the RFI, respectively. Those for the ASYNC condition were 20.0% and 40.0% in the RHI, respectively, and 30.0% and 30.0% in the RFI, respectively. This measure did not reveal a significant difference between the groups in either the RHI or RFI (hand_SYNC: *p* = 0.916; foot_SYNC: *p* = 0.711; hand_ASYNC: *p* = 0.382; foot_ASYNC: *p* = 0.820). Further, no significant difference was observed between the SYNC and ASYNC conditions in either group (LOW_fall-risk__hand: *p* = 0.070; LOW_fall-risk__foot: *p* = 0.650; HIGH_fall-risk__hand: *p* = 0.656; HIGH_fall-risk__foot: *p* = 0.179).

**Figure 3:**
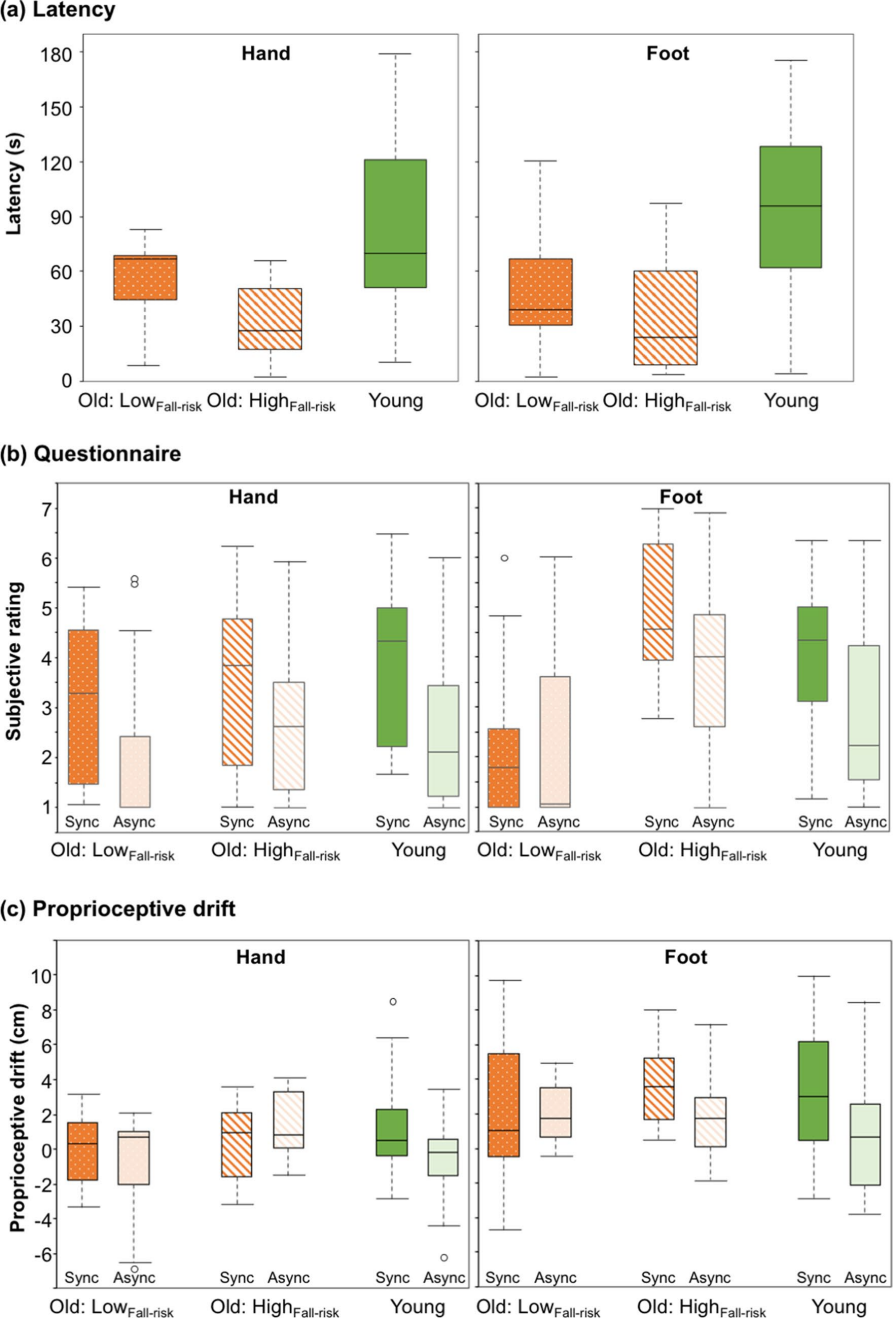
Boxplots of each measure in the high fall-risk (HIGH_fall-risk_) and low fall-risk (LOW_fall-risk_) older adult and younger adult group. (**a)** Latency in the synchronous condition. (**b**) Subjective rating of illusion in the synchronous and asynchronous conditions. The subjective rating is the average of all nine questionnaire items. (**c**) Proprioceptive drift. The origin of the proprioceptive drift measurement was the position of the real hand. Positive and negative values indicate a drift toward and away from the rubber body part, respectively. The middle line, and upper and lower limits of the box plot indicate the median, 75th, and 25th percentile. The error bars represent the maximum or minimum values where all data in the given condition fall between 1.5 times above and below the interquartile range. However, the bars represent 1.5 times above and below the interquartile range where there are outliers (blank circle) that fall beyond 1.5 times above and below the interquartile range. The data for the younger adult group are the same as in Fig. 2.

Figure 3a shows the results of latency in the RHI and RFI for the LOW_fall-risk_ and HIGH_fall-risk_ groups. The results of the younger adult group are also shown as a baseline. The Kruskal–Wallis test revealed a significant difference between the groups in the RFI (*H* = 6.443, *p* = 0.040), but not in the RHI (*H* = 5.746, *p* = 0.057). The multiple comparison revealed that the latency to perceive RFI was significantly shorter in the LOW_fall-risk_ group than in the younger adult group.

Figure 3b,c show the results of the subjective rating of illusion and proprioceptive drift. The Kruskal–Wallis tests revealed a significant difference between the groups only in the SYNC condition of the RFI in the subjective rating of illusion (hand_SYNC: *H* = 1.165, *p* = 0.558; foot_SYNC: *H* = 10.976, *p* = 0.004; hand_ASYNC: *H* = 2.751, *p* = 0.253; foot_ASYNC: *H* = 4.155, *p* = 0.125). The multiple comparison revealed that the LOW_fall-risk_ group exhibited lower subjective rating of illusion scores than the other groups. Meanwhile, no significant difference between the groups was observed for the proprioceptive drift measure (hand_SYNC: *H* = 0.821, *p* = 0.663; foot_SYNC: *H* = 1.586, *p* = 0.453; hand_ASYNC: *H* = 5.189, *p* = 0.075; foot_ASYNC: *H* = 2.193, *p* = 0.334). Moreover, no effect of stroking was observed in the subjective rating of illusion (hand_ LOW_fall-risk_: *Z* = − 1.997, *p* = 0.046, *r* = 0.632; hand_ HIGH_fall-risk_: *Z* = − 1.521, *p* = 0.128, *r* = 0.481; foot_ LOW_fall-risk_: *Z* = − 0.135, *p* = 0.893, *r* = 0.043; foot_ HIGH_fall-risk_: *Z* = − 1.120, *p* = 0.263, *r* = 0.354) or proprioceptive drift (hand_ LOW_fall-risk_: *Z* = − 0.357, *p* = 0.721, *r* = 0.112; hand_ HIGH_fall-risk_: *Z* = 0.866, *p* = 0.386, *r* = 0.274; foot_ LOW_fall-risk_: *Z* = 0.153, *p* = 0.878, *r* = 0.048; foot_ HIGH_fall-risk_: *Z* = − 1.376, *p* = 0.169, *r* = 0.435).

## Discussion

Using the RHI and RFI, this study investigated how multisensory integration that is involved in body perception can change with increasing age and with different body parts; we also examined whether it is associated with the risk of falling in older adults. The primary measures in the study were latency, subjective rating of illusions (the ownership, localization, and agency subscales; and the whole experience, which was defined as the average of the three preceding subscales), and proprioceptive drift.

Regarding the RHI, stronger illusions occurred in the SYNC condition than in the ASYNC condition, irrespective of the age group; although this was not observed for the proprioceptive drift measure among the older adults. Furthermore, no significant difference was observed between the younger and older adults for proprioceptive drift. As such, our study replicated almost all findings observed in previous studies^5–8^. In contrast, in the RFI, the effect of the stroking was observed in all measures among the younger adults; in the older adults, it was observed only in the measure of percentage occurrence of illusion.

The comparison among the high and low fall-risk older adults and younger adults revealed intriguing results. In the RFI, older adults with a lower risk of falling had a weaker subjective rating of the illusion, compared to those with a higher risk of falling and the younger adults in the SYNC condition. Additionally, the subjective rating of illusion score in the SYNC condition was as low as that in the ASYNC condition. Further, older adults with a higher risk of falling exhibited shorter latencies for the RFI than the younger adults. These are in contrast with the results of the RHI, in which no difference was observed between the groups. These results suggest that aging may affect multisensory integration that is involved in body representation and that it can change depending on the stimulated body parts. Further, our results also suggest that multisensory integration is associated with the risk of falling in older adults. Below, we discuss the underlying mechanisms of our study’s findings from three points of view: the effect of aging on the multisensory integration involved in hand representation, the effect of aging on the multisensory integration involved in foot representation, and their association with the risk of falling in older adults.

### Aging and the multisensory integration of hand representation

Previous RHI studies that included middle-aged adults and those under 60 years old have reported inconsistent results. While Graham et al.^10^ showed a gradual increase in proprioceptive drift with advancing age, Marotta et al.^6^ demonstrated a decrease in the subjective rating of body ownership and proprioceptive drift in a middle-aged group (44–55 years, average: 50.18 years), compared with those in younger and older adults^9^. In contrast, RHI studies with adults aged over 60 have reported no or few age-related differences in the multisensory integration involved in body perception^5–8^. Our RHI results are consistent with these latter studies.

However, one result from our study was not consistent with that of most of the previous studies. In our study, no effect of stroking was observed on the proprioceptive drift in the older adults, although it was obvious in the younger adults. Several studies have shown that subjective ratings of the RHI and proprioceptive drift are not always associated; i.e., higher subjective ratings of the RHI can occur in the absence of a shift in the perceived position of the participant’s hand, and vice versa^10,38–40^. Evidence suggests that separate and dissociable processes are involved in the proprioceptive drift (visuo-proprioceptive) and sense of ownership (visuo-tactile interactions)^41,42^. However, studies that report an absence of the shift itself (i.e., no difference from zero) are rare. One possible explanation for the lack of effect of stroking on the proprioceptive drift in the older adults may be that their proprioception might have been so unstable and variable that its effect was insufficiently strong to be detected by the measure of proprioceptive drift in our study. Further, the participants completed each condition only once in our study, which may have resulted in a relatively poor signal-to-noise ratio. However, the statistical test revealed no significant difference between the older and younger adults. This suggests that the proprioceptive sense in the older adults was not much worse than that of the younger adults in our study. Riemer et al.^8^ asked their older participants to indicate the perceived position of the index finger of their own right hand five times in a sequence after the induction of the RHI. They showed that there were no differences in the variance in the lateral direction between the younger and older adults, indicating that the older adults in their study exhibited as stable a sense of body position as the younger adults.

Another possibility may have been related to our method of measuring proprioceptive drift. We used a sliding marker technique, in which an LED slowly moved along a straight line in a dark room; the participants were asked to verbally indicate when they felt that the LED device was over their middle digit. We adapted this method so that the participants indicated the perceived position of their digit in almost the same manner for their hand and foot, without using other visual cues to specify the positions. However, this procedure is dependent on a stable and accurate binocular vision, which was not assessed in our study. This may have been disadvantageous for older adults, whose vision (especially near-vision) was significantly poorer than that of the younger adults. This may have led to unstable responses, resulting in an absence of the group effect during the proprioceptive drift measure. A greater number of responses per condition should have been obtained to investigate the stability of the participants’ responses, as performed in the studies by Campos et al.^5^ and Riemer et al.^8^. Therefore, more data are required to discern the underlying mechanisms regarding the absence of the proprioceptive drift in older adults, as observed in our study.

### Aging and the multisensory integration of the foot representation

One of our novel findings was that the effect of aging on the multisensory integration involved in the foot representation was not consistent with that of the hand representation. While there were no significant group differences (younger vs. older adults) in either the SYNC or ASYNC conditions, the effect of stroking was pronounced in all measures in the younger adults, whereas it was absent in the older adults, except in the percentage occurrence of illusions. This is in contrast with the results of the RHI; the effect of stroking was pronounced in both groups, except in the proprioceptive drift measure. While some studies with healthy younger adults have shown similar multisensory integration mechanisms for the hand and foot^43,44^, others have suggested that there may be some differences in these mechanisms between the hands and feet^17,45^. Our study provides evidence that supports the latter literature.

We speculated that the lack of effect of stroking in the RFI might be linked to the prolonged temporal windows of multisensory stimuli integration. Several studies using audio-visual stimuli have shown that the temporal window of integration is typically larger in older adults than in younger adults^21,46^. Poliakoff et al.^47^ have reported that the visuo-tactile temporal processing of the hand deteriorated with advanced age. However, it is not clear how advancing age affects the temporal window of the visuo-tactile and visuo-proprioceptive interactions that are related to the RFI. Shimada et al.^48^ have reported that a temporal discrepancy of less than 300 ms between the visual and tactile stimuli was necessary to induce a strong RHI, and that the maximum delay for a significant RHI to occur was 500 ms. Additionally, Costantini and colleagues^49^ have demonstrated that the temporal window of integration in the RHI depends on the individual’s sensitivity to perceive the temporal gap. In our experimental setting, the delay between the visual and tactile stimuli was approximately 1000 ms. Thus, our results suggest that the older adults’ temporal window of the visuo-tactile and visuo-proprioceptive interactions in the foot representation is prolonged to 1000 ms or longer, and that it differs from that in the hand representation.

### Association between the illusions and older adults’ risk of falling

Another novel finding of our study was that the RFI was closely associated with the risk of falling in the older adults, as assessed by the TUG test. There was a clear difference between the groups in the subjective rating of illusion in the foot SYNC condition. Older adults with a lower risk of falling exhibited a weaker RFI than the younger adults and those with a higher risk of falling. This was specifically observed in the RFI but not in the RHI. Thus, it is likely that the mechanisms of multisensory integration in foot representation differ between the HIGH_fall-risk_ and LOW_fall-risk_ groups.

Previous studies that have investigated age-related changes in visuo-tactile interactions have shown that multisensory interactions are enhanced in older adults^47,50,51^. This is reported to be especially pronounced in older adults with degraded postural stability^23^, and decreased gait and balance functions^25^. The result of the RFI in the latency measure in the HIGH_fall-risk_ group may be closely linked to these previous results. However, the mechanism underlying this phenomenon remains unexplained. It has been suggested that optimal multisensory integration involves the integration of information from different sensory modalities, based on the relative reliability of their cues, using the maximum likelihood estimation^18^. In this study, no significant differences were observed between the HIGH_fall-risk_ and LOW_fall-risk_ groups in either visual acuity or tactile sensitivity, although these senses were clearly degraded in the older adults, compared to those in the younger adults. Furthermore, the variability of the proprioceptive sense of the foot did not significantly differ between the HIGH_fall-risk_ and LOW_fall-risk_ groups. Thus, it does not seem that the HIGH_fall-risk_ and LOW_fall-risk_ groups differ in the relative reliability of the sensory modality cues, as far as the indices used in our study indicate. This is consistent with the data in the study by Teramoto et al.^25^; they reported that older adults with poor gait and balance functions exhibited enhanced visuotactile interaction, compared with those with relatively good gait and balance functions. However, no significant differences were observed in visual acuity, tactile sensitivity, and visual and tactile reaction times between these groups. Thus, such a bottom-up account would not fully explain the difference between the HIGH_fall-risk_ and LOW_fall-risk_ groups.

It may be that the LOW_fall-risk_ group ceases to rely on such degraded and unreliable visual information; conversely, the HIGH_fall-risk_ group may continue to rely on visual signals as much as or more than younger adults normally do, despite their visual sensitivity or precision being degraded. Some studies have suggested that a decline in inhibitory or attention mechanisms, in which task-relevant information is efficiently selected, influences the performance in older adults^21,51^. We assessed these abilities using the TMT-A and TMT-B and observed that the HIGH_fall-risk_ group exhibited a trend of lower TMT-A scores than the LOW_fall-risk_ group. This difference might have influenced how the illusions occurred. However, it remains unexplained why the inhibition of visual information in the LOW_fall-risk_ group appeared only in the RFI but not in the RHI.

According to the Bayesian framework, prior knowledge also contributes to the final perception, in addition to the bottom-up sensory signals^52^. The degree to which prior knowledge contributes to the final perception relative to the sensory input is assumed to be determined by the relative reliability. In current literature, prior knowledge includes the cue reliability of each sensory modality, cue correspondence, and internal models of how each body part looks and moves. It is possible that either prior knowledge or its reliability may differ depending on the body part. Previous studies have suggested that prior knowledge can be updated or recalibrated through learning the statistical characteristics of the surrounding environment^53,54^. A recent study has demonstrated that after prolonged (one week) immobilization of one hand, the strength of the RHI was increased for that hand and decreased for the other^55^. This suggests that daily physical activity can affect the updating of prior knowledge or its reliability. Considering that the LOW_fall-risk_ group exhibited a significantly higher amount of daily physical activity than the HIGH_fall-risk_ group, the amount of daily physical activity and the sensory feedback from it may play an important role.

This study has several limitations. First, we used only the TUG test to assess the risk of falling in the older adults. The TUG test is a common tool in clinical situations to identify older adults who are at risk of falling^32,33^. However, to increase the reliability and generalize the findings of our study, it is necessary to conduct further studies using other fall-risk assessment tools such as postural stability tests^56^. Second, there were slight differences in the results of the RFI among the measures. Specifically, while the latency measure showed shorter onset times taken to perceive the illusion in the HIGH_fall-risk_ group than in the younger adult group, the subjective rating measure showed smaller RFI in the LOW_fall-risk_ group than in the other groups. Moreover, the proprioceptive drift measure did not show any group difference in the RFI. While this may be related to the third concern described below, there is a possibility that the different measures may reflect different aspects of the underlying mechanisms in body perception. The third concerns statistical power. Although the sample size for the comparison between younger and older groups in our study was comparable to that in several previous studies^5,6,8^, the analyses on the differences between the HIGH_fall-risk_ and LOW_fall-risk_ groups were performed on relatively small sample sizes; this might have resulted in the appearance of only statistically strong effects. We must address these issues in future studies to make more definitive conclusions.

## Conclusions

This study used the RHI and RFI to investigate how multisensory integration involved in body perception changes with increasing age and for different body parts, and whether it is associated with the risk of falling in older adults. In agreement with previous studies that examined older adults over 65 years old, almost all of the results in the RHI were consistent between the younger and older adults. However, in the RFI, older adults with a lower risk of falling exhibited the weakest subjective rating of illusion in the SYNC condition, which was as low as that in the ASYNC condition. In contrast, those with a higher risk of falling more rapidly experienced the illusion than the other adults and reported a subjective rating of illusion that was as strong as that reported by the younger adult group in the SYNC condition. These results suggest that the way in which older adults integrate multisensory information can change, depending on the stimulated body parts, and that the risk of falling affects multisensory integration in older adults.

## Data Availability

The datasets generated and analyzed during the current study are available from the corresponding author on reasonable request.
